# Social pediatrics: weaving horizontal and vertical threads through pediatric residency

**DOI:** 10.1186/s12909-016-0845-4

**Published:** 2017-01-13

**Authors:** Meta van den Heuvel, Maria Athina Tina Martimianakis, Rebecca Levy, Adelle Atkinson, Elizabeth Ford-Jones, Michelle Shouldice

**Affiliations:** 1 Department of Paediatrics, University of Toronto, Toronto, ON Canada; 2 Division of Paediatric Medicine, Hospital for Sick Children, M5G 1X8 555 University Avenue, Toronto, ON Canada; 3 Peter Gilgan Center for Research and Learning, 10th floor, room 10.9830, 686 Bay Street, M5G0A4 Toronto, ON Canada

**Keywords:** Social pediatrics, Social determinants of health, Curriculum map, Pediatric resident education, Competencies

## Abstract

**Background:**

Social pediatrics teaches pediatric residents how to understand disease within their patients’ social, environmental and political contexts. It’s an essential component of pediatric residency training; however there is very little literature that addresses how such a broad-ranging topic can be taught effectively. The aim of this study was to determine and characterize social pediatric education in our pediatric residency training in order to identify strengths and gaps.

**Methods:**

A social pediatrics curriculum map was developed, attending to 3 different dimensions: (1) the intended curriculum as prescribed by the Objectives of Training for Pediatrics of the Royal College of Physicians and Surgeons of Canada (RCPSC), (2) the formal curriculum defined by rotation-specific learning objectives, and (3) the informal/hidden curriculum as reflected in resident and teacher experiences and perceptions.

**Results:**

Forty-one social pediatric learning objectives were extracted from the RCPSC Objectives of Training for Pediatrics, most were listed in the Medical Expert (51%) and Health Advocate competencies (24%). Almost all RCPSC social pediatric learning objectives were identified in more than one rotation and/or seminar. Adolescent Medicine (29.2%), Pediatric Ambulatory Medicine (26.2%) and Developmental Pediatrics (25%) listed the highest proportion of social pediatric learning objectives. Four (10%) RCPSC social pediatric objectives were not explicitly named within learning objectives of the formal curriculum. The informal curriculum revealed that both teachers and residents viewed social pediatrics as integral to all clinical encounters. Perceived barriers to teaching and learning of social pediatrics included time constraints, particularly in a tertiary care environment, and the value of social pediatrics relative to medical expert knowledge.

**Conclusions:**

Despite the lack of an explicit thematic presentation of social pediatric learning objectives by the Royal College and residency training program, social pediatric topics are integrated, taught and learned throughout the entire curriculum. Special attention needs to be given to the hidden curriculum and system barriers that may impede social pediatric education.

## Background

*“Every disease has two causes one pathophysiological and one political” (CITATION)* [1]*.* Nowadays this quote from Rudolf Virchow (1821–1902) seems truer than ever. An increasing body of research has shown that life success and long-term well-being are results of the conditions in which children live, grow, learn and play [2–6]. A basic scaffolding of health, education, and family support is needed to achieve optimal lifelong well-being [2, 3, 7]. As the understanding and the impact of biological, behavioral, economic, cultural, social, political and physical environments on healthy development deepens and expands, the long standing role of pediatricians and medical educators in promoting the bio-psycho-social well-being of all children must also evolve [4]. In the Canadian setting, social pediatrics has been defined as “the orientation of pediatric training and practice to include the study of the social determinants of health and to develop multidisciplinary strategies to moderate their protean effects on child health and disease outcomes” [8]. Social pediatrics teaches pediatric residents how to understand disease within their patients’ social, environmental and political contexts. The International Society for Social Pediatrics and Child Health (ISSOP) uses a broader definition of social pediatrics as a "global, holistic, and multidisciplinary approach to child health: it considers the health of the physical, mental, and social dimensions of child health and development as well as care, prevention and promotion of health and quality of life" [9].

While social pediatrics is an essential component of pediatric residency training, there is very little literature which addresses how such a broad-ranging topic can be taught effectively. Published reports of pedagogical approaches to social pediatrics including electives [8, 10], community rotations [11], advocacy projects [12] and lectures [13, 14], suggest that to teach explicitly about the social determinants of health, the evidence for how social, political, cultural and economic conditions influence health should be embedded as a vertical thread running throughout all parts of curriculum [15], rather than in discrete educational experiences. It has also been a well-established tenet of medical education that learning in context is essential, so that learners should have the opportunity to experience first-hand how the social determinants of health impact health outcomes [8, 10, 13, 16].

We undertook a comprehensive exploration of how social pediatrics is being taught, and how this teaching is experienced by residents, in our core pediatric training program at the University of Toronto. We set out to create a social pediatric curriculum map. A curriculum map is an educational tool that details learning objectives (intended curriculum), what is taught (the learning opportunities representing the formal curriculum), when it is taught (curriculum sequence) and assessment of learning outcomes [17, 18]. For our study we chose to evaluate both the intended and formal curriculum of social pediatric education. Since social pediatrics is often viewed as less important than biomedical science and technology in medical education, we also explored the “hidden curriculum” of social paediatrics [19]. The overall aim of this study was to determine and characterize social pediatric education in our pediatric residency training in order to understand strengths and gaps. Lessons learned from this study will inform future planning on how an integral curricular concept such as social pediatrics can be effectively woven through medical training.

## Methods

### Setting

The Hospital for Sick Children is affiliated with the University of Toronto and is the largest academic pediatric hospital of Canada. Approximately 75 pediatric residents are trained in inpatient, outpatient and community settings.

### Curriculum mapping

We approached the curriculum mapping, attending to 3 different dimensions of curriculum: (1) the intended curriculum; consisting of the learning objectives as prescribed by prescribed by the Objectives of Training for Pediatrics of the Royal College of Physicians and Surgeons of Canada (RCPSC) (2) the formal curriculum defined by local rotation- and seminar specific learning objectives, and (3) the informal (“hidden”) curriculum as reflected in student and teacher experiences, opinions and perceptions.

### Intended curriculum

Pediatric competencies to be acquired during residency training are prescribed in the "Objectives of Training in Pediatrics" published by the Royal College of Physicians and Surgeons of Canada (RCPSC) [20]. In this document, competencies are framed through the CanMEDS roles [21]. The CanMEDS framework outlines seven roles for physicians, including: Medical Expert, Communicator, Collaborator, Manager, Health Advocate, Scholar, and Professional. In the Objectives of Training in Pediatrics, social pediatric competencies are not described within a separate category under Medical Expert (like for example "neonatology"), but are rather dispersed throughout other categories and roles. In order to identify social pediatrics objectives, five authors (MH, AA, LFJ, MS, MAM) with expertise in social pediatrics and medical education independently reviewed the document and extracted social pediatric learning objectives. The ISSOP definition of social pediatrics was used for the purposes of this study [9]. Authors utilized identifiers associated with social paediatrics, including the following terms: "(psycho) social factors, (psycho) social development, biopsychosocial model, social history, social support and (social) determinants. Group meetings were held to discuss the individually identified objectives and reach consensus (Table 1).

**Table 1:** RCPSC social pediatric learning objectives (intended curriculum) mapped to formal curriculum

Number^a^	Royal College Social Pediatric Competency	Formal curriculum^b^
COMMUNICATOR		
C1.4	Listen effectively; obtain and synthesize relevant history from patients, families and communities	Developmental Pediatrics, NICU, seminar
C2.1	Gather information about a disease, but also about a patient's beliefs, concerns, expectations and illness experience	Oncology, Adolescent Medicine
C2.1.2	Give close attention to impact of such factors as age, gender, disability, ethno-cultural background, social support and emotional influences on a patients’ illness	Oncology, Pediatric Perinatology, seminar
C2.2	Seek out and synthesize relevant information from other sources, such as a patient's family, caregivers and other professionals	Developmental Pediatrics
C2.2.2	Demonstrate an appreciation of the parent's perspective of and concerns for a child's health and the impact of a child's illness on family relationships	Oncology
C4.1	Identify and explore problems to be addressed from a patient encounter effectively, including the patient's context, responses, concerns and preferences	Adolescent Medicine, Emergency Medicine, seminar
C4.2	Respect diversity and difference, including but not limited to the impact of age, gender, abilities, religion, language and cultural beliefs…	Developmental Pediatrics, seminar
COLLABORATOR		
Col1.11	Collaborate with teachers, social workers, community leaders, child protection workers and other professionals	Pediatric Medicine-PGY3/1, Oncology, seminar
HEALTH ADVOCATE		
H1.2	Identify opportunities for advocacy, health promotion and disease prevention with individuals to whom they provide care	Adolescent Medicine, Pediatric Perinatology, Pediatric Ambulatory Medicine, Developmental Pediatrics, Respiratory Medicine, seminar
H2.2	Identify opportunities for advocacy, health promotion and disease prevention in the communities that they serve	Pediatric Ambulatory Medicine, Developmental Pediatrics, Pediatric Perinatology, seminar
H2.3	Appreciate the possibility of competing interests between the communities served and other populations	Pediatric Ambulatory Medicine, seminar
H3.1	Identify the determinants of health of children; including barriers to access to care and resources	Developmental Pediatrics, Pediatric Perinatology, seminar
H3.2	Identify vulnerable or marginalized populations within those served and respond appropriately	Adolescent Medicine, seminar
H4.1	Describe an approach to implementing a change in a determinant of health of children	seminar
H4.2	Describe how public policy impacts on child health	Respiratory Medicine, Pediatric Perinatology, Cardiology, seminar
H4.4	Describe the ethical and professional issues inherent in health advocacy…	Pediatric Ambulatory Medicine, Endocrinology, Respiratory Medicine, seminar
H4.5	Appreciate the possibility of conflict inherent in their role as health advocate for a patient or community with that of manager or gatekeeper	seminar
H4.6	Describe the role of the medical profession in advocating collectively for health and patient safety	seminar
MEDICAL EXPERT		
M2.1.12.7	Social, familial and personal effects of childhood cancer	Oncology, seminar
M2.1.14.4	Demographic, medical and psychosocial factors which influence perinatal mortality and morbidity	Pediatric Perinatology, seminar
M2.1.22.3	Availability of and access to community-based mental health resources	seminar
M2.1.22.4	Biological, psychological and socioeconomic factors affecting mental health	Adolescent Medicine, seminar
M2.1.22.5	Impact on child well-being of having a parent with mental illness or substance abuse	seminar
M2.1.24.1	Social factors placing children at risk	Adolescent Medicine, Pediatric Ambulatory Medicine, seminar
M2.1.24.2	Impact of violence on health	Pediatric Ambulatory Medicine, Adolescent Medicine, seminar
M2.1.24.3	Health problems consequent to maltreatment/neglect	Pediatric Ambulatory Medicine, Adolescent Medicine
M2.1.24.4	Laws relating to child protection	Pediatric Ambulatory Medicine, seminar
M2.1.24.5	Professional requirements in managing victims of maltreatment/neglect including mandatory reporting	Pediatric Ambulatory Medicine, Adolescent Medicine
M2.1.3.2	Adolescent & Society; influencing factors, heterogeneity, subcultures	Adolescent Medicine
M2.1.7.2	Biological and psychosocial factors affecting development and behavior	Pediatric Ambulatory Medicine, Adolescent Medicine, Developmental Pediatrics, seminar
M5.1.13.3	Assessment of adolescent using HEEADS format (Home, Education, Eating, Activity, Drugs, Sexuality, Suicide)	Adolescent Medicine
M5.1.16.2	Counselling parents on normal growth, development and behavior; attention to available community support and resources	Pediatric Perinatology, Developmental Pediatrics, seminar
M5.1.30.1	Gather child maltreatment evidence appropriately including documentation and specimen collection	seminar
M3.2.4	Identify all other important information from the past history; and social history	seminar
M3.1	Identify and explore issues to be addressed in patient encounter, including the patient’s and family’s context and preferences	seminar
M3.2	Elicit a history that is relevant, clear, concise and accurate to context and preferences	seminar
PROFESSIONAL		
P1.3.4	Demonstrate knowledge of the legal and ethical codes of professional behavior… reporting suspected child or sexual abuse	Endocrinology, Pediatric Perinatology, seminar

^a^Reference number “Objectives of training in Pediatrics" published by the Royal College of Physicians and Surgeons of Canada (RCPSC) [20]

^b^Rotation or core educational seminar where learning objective was found

### Formal curriculum

The pediatric residency program at our university consists of 31 mandatory rotations and 6 elective blocks over three core years. Each rotation has its own learning objectives linked to assessment which are published in "In-Training Evaluation Reports" (ITERs). Each rotation’s ITER was assessed by two authors (all authors looked at different ITERs) and mapped these to the RCPSC social pediatric learning objectives. In addition one author (MH) examined the learning objectives of the core educational seminar series, and mapped these to the RCPSC social pediatric learning objectives (Table 1).

### Informal curriculum

Surveys were used as the primary tool to identify and characterize teacher and learner perceptions, experiences and opinions related to social pediatrics education at our institution. Online surveys were distributed via email to all residents enrolled in the training program (*n* = 75). Similar surveys were distributed to all physician faculty appointed at the primary affiliated pediatric teaching hospital (*n* = 170), as all staff physicians at our institution are mandated to teach residents in some capacity. Three email reminders were sent over a 2-month window. The surveys consisted of closed and open-ended questions. Results were collected anonymously using surveymonkey.com, an online survey distribution tool.

Survey questions were generated from review of relevant literature, the identified social pediatric learning objectives, and through discussion in our research team meetings. Some of the topics addressed included: where in each rotation social pediatric education took place, the perceived teaching and learning of social pediatric concepts, awareness of formal learning objectives and perceived barriers in social pediatric education. Quantitative data were analyzed using descriptive statistics, and open-ended responses were analyzed using a combination of meaning condensation to represent perceptions and experiences of teachers and learners, and meaning categorization to distil key points and generate representative thematic categories.

## Results

### Intended curriculum

**Fig. 1:**
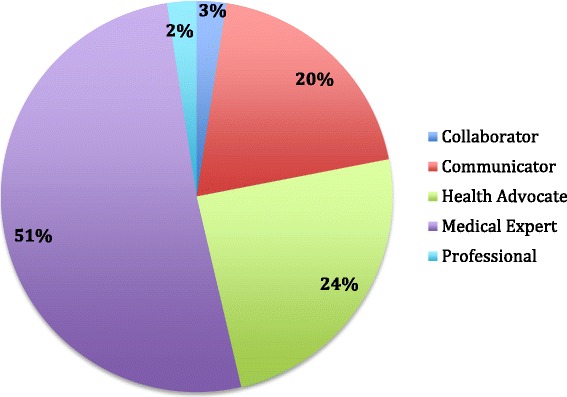
CanMEDS Competencies in the RPSC social pediatric learning objectives

Forty-one social pediatric learning objectives were extracted from the RCPSC Objectives of Training for Pediatrics, within five CanMEDS (Communicator, Collaborator, Health Advocate, Professional, Medical Expert). No specific social pediatric learning objectives were identified within the Manager and Scholar competencies. Most were identified in the Medical Expert (51%) and Health Advocate (24%) roles (Fig. 1).

### Formal curriculum

**Fig. 2:**
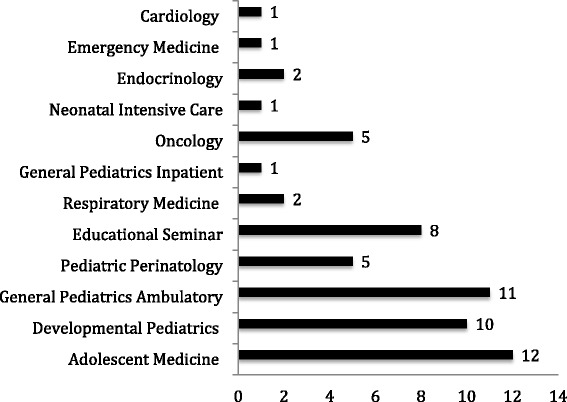
Quantity and distribution of the RPSC social pediatric learning objectives in different rotations

Table 1 shows the formal curriculum rotation- and core educational specific learning objectives, mapped to the intended curriculum (RCPSC social pediatric objectives). Almost all RCPSC social pediatric learning objectives were identified in more than one rotation and/or educational seminar. The highest proportion of RCPSC social pediatric learning objectives were listed in Adolescent Medicine (*n* =12; 29.2%), Pediatric Ambulatory Medicine (*n* =11; 26.2%) and Developmental Pediatrics (*n* = 10, 25%) (Fig. 2). Seven (12%) RCPSC social pediatric learning objectives were exclusively taught during the core educational seminar series. Four (10%) RCPSC social pediatric learning objectives were not explicitly listed in the formal curriculum (Table 2).

**Table 2:** Intended curriculum (RCPSC) learning objectives, which were not identified in the formal curriculum

CanMEDS Role	Number^a^	Royal College Competencies
Communicator	C2.1.1	Demonstrate respect for patients & families and for their values systems which may be different than pediatrician’s own values
Medical Expert	M2.1.3.6	Laws and resources in adolescence
	M2.1.18.7	Effects of chronic rheumatic diseases on physical growth and social development
	M2.1.16.4	Health implications of restricted diets, or diets determined by custom or socioeconomic situation

^a^Reference number “Objectives of training in Pediatrics" published by the Royal College of Physicians and Surgeons of Canada (RCPSC) [20]

### Informal curriculum

Surveys were collected from a total of 20 residents (response rate 27%) and 41 faculty (response rate 24%). Resident participants represented a range of training levels, and staff physicians represented a range of general and subspecialty practice (eight physicians specialized in academic paediatrics, four in emergency medicine) two in adolescent medicine, three in rheumatology, four in haematology/oncology, two in developmental paediatrics and one from all other specialities; neonatology, palliative care, endocrinology, dermatology, cardiology, nephrology, respiratory medicine, infectious diseases and child maltreatment, nine physicians did not provide their academic background).

Teachers and learners valued social pediatrics as an important component of pediatric residency training and integral to all clinical encounters. While 92.7% of staff physicians reported teaching about social pediatrics, only 52.6% of the residents felt that they had enough opportunities to learn social pediatrics in the residency program.

**Fig. 3:**
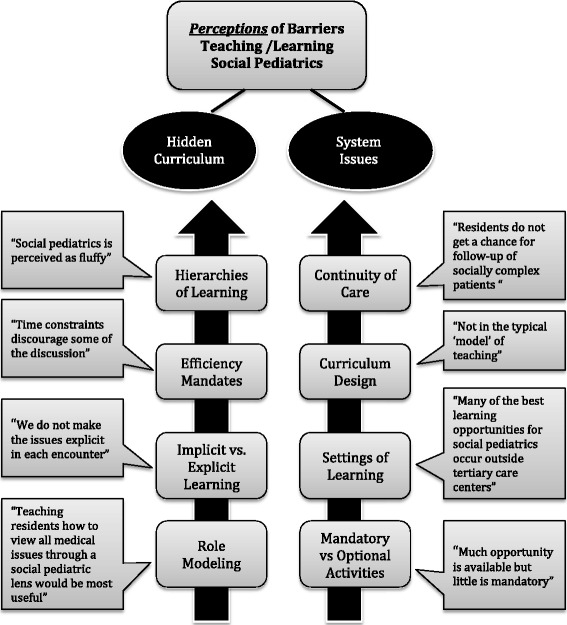
Perceived barriers to teaching and learning of social pediatrics

Multiple perceived barriers were described to limit both the teaching and learning of social pediatrics based on resident and staff survey responses. Fifty percent of staff and 68% of residents felt there were missed opportunities to teach/learn social pediatrics, attributable to these barriers. Residents and staff most frequently indicated that the “complexity of finding answers to social problems” was a barrier to social pediatrics education (79% residents; 89% staff). The perceived barriers were thematically grouped into either (A) The Hidden Curriculum or (B) Systems Issues. Fig. 3 elaborates on the perceived barriers with representative quotes from the open-ended responses of participants.

### Hidden curriculum

Social pediatrics education was commonly found to compete with other learning objectives, particularly scientific or “Medical Expert” knowledge. Furthermore, residents felt that staff over-valued teaching content related to specific medical knowledge, whereas faculty felt that residents were merely interested in getting exposed to “examinable” scientific material. Fifty-four percent of staff and 47% of residents perceived that topics discussed in social pediatrics are “time consuming” and cited a lack of time as a barrier to social pediatrics education. Respondents commonly perceived a lack of explicit objectives as limiting social pediatric education. Residents described a need for more guidance and positive role modeling from their faculty; just over half of residents (53%) perceived that lack of staff knowledge impeded social pediatrics teaching while 37% perceived staff to be disinterested in social pediatrics. Faculty described that lack of knowledge (57%) and lack of interest (27%) among staff acted as barriers to social pediatrics education.

### System issues

Another important barrier identified in the exploration of the informal curriculum of social paediatric were system issues (Fig. 3). In our program, core pediatric residents have not routinely participated in a continuity clinic, until their final year of training. A lack of continuity of care was perceived to limit the exploration of social issues, particularly in the context of isolated encounters with patients and significant time constraints related to working in inpatient units. Residents, but not staff, reported that they believed that a tertiary care hospital was not the ideal setting for social pediatrics learning. Residents perceived that other settings, such as community hospitals, offices and home visits, would potentially provide better exposure to social pediatrics learning, particularly around how to advocate for patients. Both teachers and learners felt that there were missed opportunities related to the mandatory rotations within the curriculum, but that many optional or elective opportunities were available to fill this perceived gap in their formal social pediatrics learning.

## Discussion

In this study we explored social pediatric education by creating a curriculum map which encompassed the intended, formal and informal/hidden curriculum dimensions. Our aim was to better understand how to effectively ensure that graduates from pediatric training have the skills necessary to understand disease within their patients’ social, environmental and political contexts, factors key to optimizing child and adult health outcomes.

The intended curriculum, as mandated by colleges and accreditation councils, sets the stage for the curriculum delivered through individual training programs, highlighting and emphasizing specific requirements. Examining the intended curriculum in our context, it is interesting to highlight that the RCPSC Objectives of Training in Pediatrics did not specifically define social pediatric learning objectives under a separate topic heading. This could contribute to the perception that social pediatrics is less important subject matter in terms of core medical expert knowledge, and adding to the challenge of ensuring these objectives are clearly included in the delivered curriculum.

In the formal curriculum of our training program, social pediatric learning objectives were not thematically presented in rotations or core seminar series. This reflects that the rotation-specific objectives are based upon the Royal College documents. We were able to identify all the RCPSC intended social pediatric learning objectives, except four, expressed in learning objectives of different rotations and core seminars. Given the importance of social pediatrics to all patient and family encounters, it is notable that not all social pediatric learning objectives of core content, were identified in each rotation. For example, in the Communication competency, the objective “give close attention to impact of such factors as age, gender, disability, ethno cultural background, social support and emotional influences on a patient’s illness” is arguably of key importance to weave horizontally and vertically through a residency curriculum and was represented in only two out of 12 rotations (16.7%). In another relevant example, the learning objective “social factors placing children at risk” was explicitly identified only in Adolescent Medicine and Ambulatory Pediatric Medicine, perhaps contributing to the perception that this issue is not relevant in inpatient and subspecialty care.

The evaluation of the informal curriculum revealed several perceived barriers to social pediatric learning. Hidden curriculum effects were evident in student and faculty responses. Participants perceived an implicit devaluing of social pediatric content through an over emphasis on core competencies linked to the CanMEDS Medical Expert Role. This has been previously described in the medical education literature. While it is well-accepted that being a good doctor requires far more than biomedical expertise [22], it has also been recognized that there remains “a deeply rooted tendency to consider biomedical expertise as based in “facts” and to dismiss other important areas of physician competence (communication, collaboration, professionalism) as “soft skills” that do not require similar groundings in appropriate forms of knowledge” [23]. Some of these perceptions may be unintentionally reinforced by the guiding documents of the college and residency program. There is a redesign of residency education currently underway in Canada which may address some of these issues, through a focus on direct observation of milestones and competencies guiding curriculum and assessment, “competency by design” [24].

An additional barrier perceived by both residents and staff was lack of time. Certainly, the current focus on efficiency, early discharge and optimal patient flow in academic teaching hospitals where the core of medical education often occurs, has been noted to result in more focused clinical assessments, an emphasis on acute medical issues, and less time for discussion about social aspects of clinical care despite their key role in patient outcomes [25, 26].

Additional system issues were identified as potential or perceived barriers to social pediatric education. Typical curriculum design in residency programs is predominantly a rotation system, each rotation consisting of practice in a particular setting (such as the emergency department, clinic or, inpatient unit), often prioritizing acute care or consultation. While continuity clinics, longitudinal experiences with a focus on follow-up of patients, communication and long-term responsibilities are also required by many specialties, they often form the minority of clinical experience. In our study, residents suggested that community hospitals, offices and home visits, would better expose residents to social paediatrics content and learning. It is interesting that residents perceived social pediatrics as not “fitting” in the tertiary care, academic teaching environment, despite the many social pediatric learning objectives identified in almost every rotation in our tertiary hospital.

In addition to the contribution of systems issues discussed above, educational experiences may be disconnected from objectives of training from the learner perspective and learners may identify social pediatrics only in its more extreme forms (i.e. advocacy work in the community or with identified vulnerable patient populations). Staff did not identify the setting of social pediatric learning as an important barrier. However they felt a need for more explicit, and rotation-specific social pediatric learning objectives to guide their teaching.

It has been well-accepted that assessment often drives learning in medical education [27]. In this study, both teachers and learners identified that some aspects of social pediatric education is neither mandatory, nor formally assessed. Assessing and managing the social determinants of health is a critical element of patient care that impacts long-term outcomes [28]. While physicians and medical schools are increasingly held accountable for their social roles in the community [29], and many training programs have developed educational experiences that focus on the social determinants of health, objective assessment of learners’ skills in applying the social determinants of health to the care of patients remains a challenge [28]. In our local setting, following this survey, we implemented a practice Objective Structured Clinical Examination (OSCE) station intended to assess resident performance in taking a social history. The results of the OSCE station identified social history-taking as an area needing support and a core seminar on this topic was added to the curriculum. More broadly, the move by the RCPSC to competency-based education and increased direct observation of clinical skills with identified practice milestones has the potential to support learning in this area.

### Limitations

In the process of extracting social pediatric learning objectives from the RCPSC "Objectives of Training in Pediatrics" our team did not identify any social pediatric learning objectives in the Manager CanMEDS role. Retrospectively, the learning objective “Describe the structure and function of the health care system as it relates to child health, including the roles of Pediatricians (Man 1.4)” could have been considered a social pediatric learning objective and we did not look for this learning objective in the formal curriculum. In the development of learning objectives for assessing and managing the social determinants of health, as described by Klein et al., the need to advocate for quality patient care and optimal care systems by pediatricians is also described [28]. In order to advocate for optimal health care systems, the Manager role is an important CanMEDS role in addition to the Advocacy CanMEDS role.

While our use of surveys to characterize both teacher and learner perceptions allowed us to capture overarching attitudes and perceptions, the close ended questions did not allow us to generate an in depth understanding of how these attitudes and perceptions are formed. It is likely that attention to the context of care happens frequently in contact with patients and families, even when it’s not identified as a learning objective in a specific rotation. Because we were not able to use in-depth interviews we were not able to fully explore this. Finally, the survey was not mandatory and less than one quarter of current residents and faculty took part in the study. Responses thus may reflect a self-selection bias.

## Conclusion

In this study we presented a methodology for mapping a curriculum component which runs horizontally and vertically throughout residency training. Residents should be exposed to social paediatrics so that they can learn to address the social determinants of health and appreciate the importance of the individual clinician to advocate on behalf of patients and their communities [19]. This mapping methodology was helpful in identifying key aspects relevant to social pediatric learning. The manner in which the intended curriculum competencies are articulated and categorized may influence the perceived value of an important area of competency such as social pediatrics. However despite the lack of an explicit thematic presentation of social pediatric learning objectives by the Royal College and residency training program, social pediatric topics are integrated, taught and learned throughout the entire curriculum. Our findings suggest that special attention needs to be given to the hidden curriculum and system barriers that may impede social pediatric education, in order to improve the promotion of bio-psycho-social well-being of all children.

## Abbreviations

ISSOP: The international society for social pediatrics and child health
ITERs: In-training evaluation reports
RCPSC: Royal college of physicians and surgeons of Canada
